# Giant Malignant Phyllodes Tumor with Secondary Thyroid Involvement

**DOI:** 10.3390/diseases14030114

**Published:** 2026-03-22

**Authors:** Daciana Grujic, Teodora Hoinoiu, Catalin-Alexandru Pirvu, Mihai Iliescu-Glaja, Simona Cerbu, Silviu Cristian Suciu, Daniel Pit, Cristina Marinela Oprean, Horia Cristian

**Affiliations:** 1 Department of Plastic, Reconstructive, and Burn Surgery, “Victor Babes“ University of Medicine and Pharmacy, Eftimie Murgu Square, No. 2, 300041 Timisoara, Romania; grujic.daciana@umft.ro; 2 Clinic of Plastic and Reconstructive Surgery, “Pius Brinzeu” Emergency County Hospital, Liviu Rebreanu Blvd. No. 156, 300723 Timisoara, Romania; mihai.iliescu.glaja@umft.ro (M.I.-G.); daniel.pit@umft.ro (D.P.); 3 Center for Advanced Research in Cardiovascular Pathology and Hemostaseology, “Victor Babes” University of Medicine and Pharmacy, Eftimie Murgu Square, No. 2, 300041 Timisoara, Romania; 4 Department of Clinical Skills, “Victor Babes” University of Medicine and Pharmacy, Eftimie Murgu Square, No. 2, 300041 Timisoara, Romania; 5 Department X Surgical Emergencies Clinic, “Victor Babes” University of Medicine and Pharmacy, Eftimie Murgu Square, No. 2, 300041 Timisoara, Romania; pirvu.catalin@umft.ro; 6 Doctoral School, “Victor Babes” University of Medicine and Pharmacy, Eftimie Murgu Square, No. 2, 300041 Timisoara, Romania; 7 Department XV of Orthopaedics, Traumatology, Urology and Medical Imaging, Discipline of Radiology and Medical Imaging, “Victor Babes” University of Medicine and Pharmacy, Eftimie Murgu Square, No.2, 300041 Timisoara, Romania; cerbu.simona@umft.ro; 8 Department II of Microscopic Morphology, “Victor Babes” University of Medicine and Pharmacy, Eftimie Murgu Square, No. 2, 300041 Timisoara, Romania; cristian_suciu@umft.ro; 9 Angiogenesis Research Centre, “Victor Babes” University of Medicine and Pharmacy, Eftimie Murgu Square, No. 2, 300041 Timisoara, Romania; 10 Pathology Department, “Pius Brinzeu” Emergency County Clinical Hospital Timisoara, 300723 Timisoara, Romania; 11 Department of Oncology, ONCOHELP Hospital, Ciprian Porumbescu Street No. 59, 300239 Timisoara, Romania; cristina.oprean@umft.ro; 12 ONCOMED Outpatient Unit, Department of Oncology, Ciprian Porumbescu Street No. 59, 300239 Timisoara, Romania; 13 ANAPATMOL Research Center, “Victor Babes” University of Medicine and Pharmacy, Eftimie Murgu Square, No. 2, 300041 Timisoara, Romania; 14 Department of Emergency Surgery, “Victor Babes” University of Medicine and Pharmacy, Eftimie Murgu Square, No. 2, 300041 Timisoara, Romania; cristian.horia@umft.ro

**Keywords:** phyllodes tumor, cervical metastasis, tracheal compression, sarcomatoid, mastectomy

## Abstract

**Background/Objectives**: Phyllodes tumors are rare fibroepithelial neoplasms of the breast, accounting for less than 1% of all breast tumors and approximately 2–3% of breast fibroepithelial tumors. They can be benign, borderline, or malignant, depending on cellular atypia and stromal invasion. Although most display local behavior, malignant forms can metastasize hematogenously, most frequently to the lungs, bones, and liver, with lymph node metastases being exceptional. **Case Presentation:** We analyzed the case of a 47-year-old woman with a phyllodes breast tumor weighing approximately 5 kg. The tumor evolved slowly over five years, followed by accelerated growth, cutaneous necrosis, superinfection, and severe anemia. Total mastectomy was performed, and histopathological examination confirmed the diagnosis of a malignant phyllodes tumor. Five months after surgery and adjuvant radiotherapy, the patient developed laterocervical metastases with thyroid invasion and life-threatening tracheal compression, an extremely rare presentation for this type of tumor in adults. **Discussion/Conclusions:** This case illustrates the aggressive and unpredictable behavior of malignant phyllodes tumors and underscores the necessity of careful oncological monitoring and a multidisciplinary approach, given their potential for dissemination to unusual locations.

## 1. Introduction

Phyllodes tumors (PTs) are rare mammary gland tumors that were first described by Johannes Müller in 1838 [1]. They arise from the stromal component of the breast and exhibit a biphasic architecture comprising epithelial and stromal elements [2].

The World Health Organization (WHO, 2020) classifies these tumors into three categories: benign, borderline, and malignant [3]. This classification is based on histopathological features, such as tumor border, stromal cellularity, stromal atypia, mitotic activity, stromal overgrowth, and malignant heterologous elements [3,4].

The incidence of malignant phyllodes tumors is estimated to be approximately 0.3–0.5 cases per 100,000 women per year, and they predominantly affect women aged between 35 and 55 years [5]. The main pathway for metastasis is hematogenous, with frequent spread to the lungs (66%), bones (28%), and liver (9%) [6].

Although nodal metastases are rare, they have been documented in the literature, including cases involving axillary and pulmonary nodes [7,8,9,10,11]. Involvement of the trachea and thyroid is extremely rare, with fewer than ten reported cases worldwide [5,8,12].

The clinical behavior and evolution of these tumors have been analyzed in several clinicopathological studies [13].

The present case illustrates the atypical progression of a malignant phyllodes tumor, which, after radical surgical treatment and adjuvant therapy, developed massive cervical metastasis with tracheal compression and invasion into the thyroid, requiring complex surgical intervention.

## 2. Case Presentation

**Figure 1:**
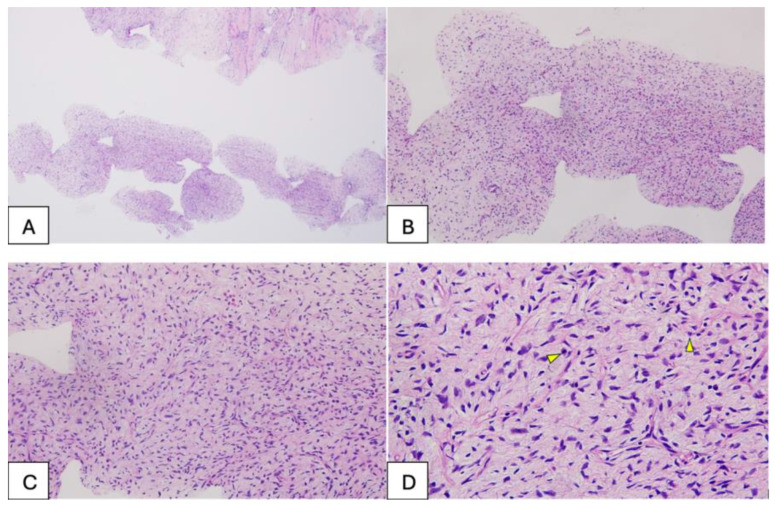
(**A**,**B**)—Tissue samples obtained from the breast tumor via breast biopsy, approximately 1 mm thick, consisting of a stromal component with variable cellularity; no epithelial structures were observed in the examined samples. (**C**,**D**)—Detail of the tumor component with mesenchymal features, showing mild to moderate cellularity, with focal nuclear overlapping and evident nuclear atypia, and mitotic activity present (indicated by the arrow). The magnification times: (**A**) (×4), (**B**) (×10), (**C**) (×20), and (**D**) (×40).

We present the case of a 47-year-old woman with cachexia (body mass index [BMI], 14.71), who had no family history of neoplastic disease and was diagnosed with an ulcerated malignant phyllodes tumor of the left breast through imaging (breast ultrasound) and core needle biopsy, weighing approximately 5 kg and with long-term evolution (Table S1, Supplementary Materials). Core needle biopsy of the breast mass revealed mesenchymal proliferation with variable cellularity and mild-to-moderate cytologic atypia within loose stroma. Immunohistochemistry showed negativity for p63, CK5, and pan-cytokeratin, with strong CD34 positivity in tumor cells, suggesting a fibroepithelial lesion consistent with a phyllodes tumor (B3 category) (Figure 1). This study was approved by the Ethics Committee of the Pius Brînzeu County Emergency Clinical Hospital in Timișoara (no. 575/16.10.2025). Written informed consent was obtained from the patient for the use of photographs and video recordings for medical education and publication purposes.

**Figure 2:**
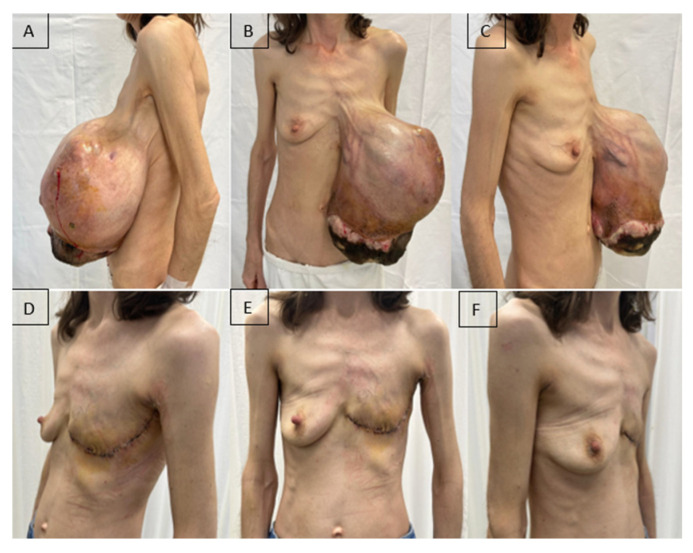
A patient with a giant malignant phyllodes tumor of the left breast (5.2 kg). (**A**–**C**): Preoperative aspect; (**D**–**F**): Fifteen days after radical mastectomy.

At the time of initial hospital admission, the patient was cachectic, with a relatively good general condition but severe anemia (Hb = 3.8 g/dL), reactive thrombocytosis (1130.7 × 10^3^/µL), and an inflammatory syndrome associated with low total serum protein and albumin levels. Local examination revealed a markedly enlarged left breast with ptosis due to excessive weight, areas of necrosis at the lower pole, and local superinfection (Figure 2A–C).

The timing of surgery was determined by correction of anemia (Hb = 8.33 g/dL), platelet count (587 × 10^3^/µL), serum protein levels, and initiation of antibiotic therapy according to the antibiogram (wound culture: *Stenotrophomonas maltophilia* and *Klebsiella oxytoca* sensitive to trimethoprim–sulfamethoxazole). The patient underwent left mastectomy with lymphadenectomy of Berg’s levels I and II under general anesthesia. Although axillary lymph node metastases are rare in phyllodes tumors, axillary lymph node dissection may be considered in the presence of clinically suspicious lymphadenopathy. In our case, the patient presented with a large and painful axillary adenopathic block, which raised the suspicion of nodal involvement and justified the decision to perform axillary lymphadenectomy at the time of the initial surgery. The postoperative course was favorable, with improvements in both the general condition and laboratory parameters (Figure 2D–F).

**Figure 3:**
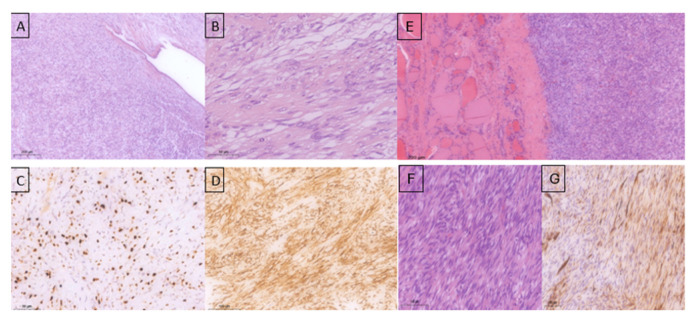
Histopathological examination of the specimen—left breast mastectomy: (**A**)—mammary gland involved by a fibroepithelial proliferation (primary tumor) with predominance of the stromal component showing both pushing and infiltrative growth patterns (HE stain); (**B**)—the tumor stroma is densely cellular, composed of spindle-shaped cells arranged in variably oriented bundles with moderate to marked nuclear atypia in places, nucleoli present, high mitotic activity (HE stain); (**C**)—Ki-67 approximately 40% (hotspot); (**D**)—CD-34 positive in a majority of tumor cells (primary tumor). (**E**)—Tumor proliferation with mesenchymal features (spindle-shaped cells) involving the thyroid parenchyma. (**F**)—Tumor with a fascicular pattern, densely cellular, showing mild-to-moderate nuclear pleomorphism, and prominent mitotic activity. (**G**)—CD34 immunostaining demonstrating heterogeneous expression within the tumor component of the thyroid parenchyma.

Histopathological examination confirmed the diagnosis of a malignant phyllodes tumor with ulceration involving the entire left breast, with associated myxoid degeneration and focal necrosis. The pathological staging was pT4aN0 (0/6). The tumor exhibited high mitotic activity (15 mitoses/10 high-power fields [HPF]), a Ki-67 proliferation index of 40%, infiltrative margins, marked stromal cellularity, moderate with focal severe nuclear atypia, and prominent stromal overgrowth. The axillary lymph nodes showed reactive inflammatory changes (Figure 3 A–D).

**Figure 4:**
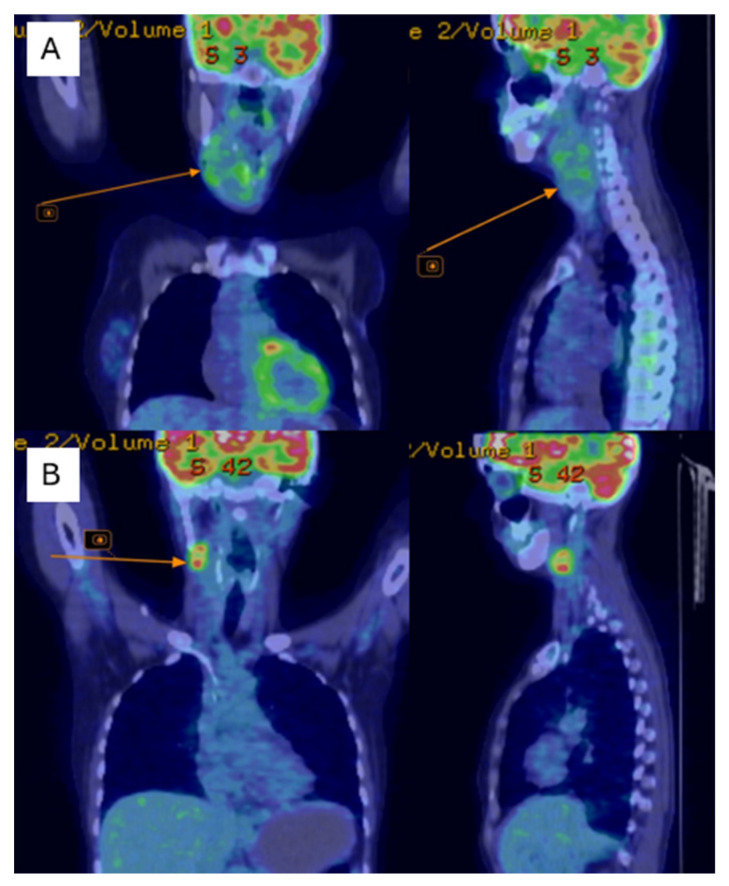
PET-CT shows of the right jugular–carotid adenopathic mass (**A**)—before chemotherapy, (**B**)—after chemotherapy.

Postoperatively, the patient underwent adjuvant radiotherapy (total dose, 50 Gy in 25 fractions) to the left chest wall using a Halcyon linear accelerator, which was well-tolerated. Three months after radiotherapy, the patient developed dysphonia, and a subcutaneous tumor mass was observed in the right lateral cervical region. Cervical ultrasonography revealed immobile right lateral cervical lymphadenopathy measuring approximately 4 × 4 cm, with a normal-sized thyroid gland. Cervical magnetic resonance imaging (MRI) revealed a bilobed malignant tumor mass in the right laterocervical region, with invasion of the right thyroid cartilage and partial involvement of the right vocal cord. PET-CT showed metabolically active secondary lesions in the right lateral cervical lymph nodes and in the right lower pulmonary lobe (Figure 4A).

A core needle biopsy of the laterocervical mass revealed sarcomatoid lymphonodular metastasis originating from the breast tumor. A Port-a-Cath central venous catheter was placed, and LT-AIM chemotherapy (adriamycin, ifosfamide, mesna) was initiated for six cycles over four months, with treatment delays due to hematological deficiencies and liver dysfunction.

**Figure 5:**
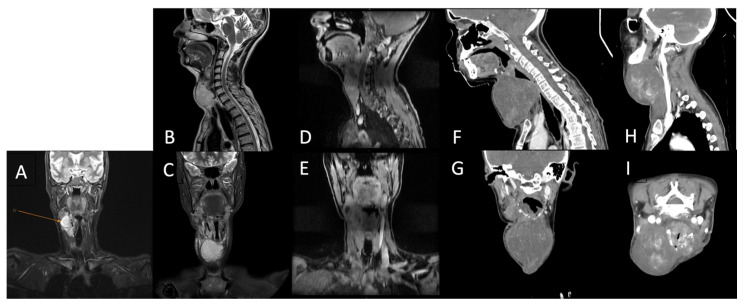
Cervical region using MRI (**A**)—After chemotherapy (5 months post-surgery); (**B**,**C**)—after adjuvant chemotherapy; (**D**,**E**)—after adjuvant radiotherapy; (**F**,**G**)—After first round of palliative chemotherapy; (**H**,**I**)—after second round of palliative chemotherapy.

Follow-up cervical PET-CT (Figure 4B) and MRI (Figure 5A–C) showed a significant reduction in the size of the right jugular–carotid adenopathic mass, with residual lymphadenopathy and a favorable pulmonary response. No new metabolically active lesions were observed.

Subsequently, the patient underwent re-irradiation (60 Gy in 30 fractions to the right laterocervical lymph nodes and 50 Gy in 25 fractions to the left laterocervical lymph nodes). Despite treatment, cervical MRI revealed rapid progression of metastatic disease, with marked enlargement of adenopathy infiltrating the thyroid cartilage, partially encasing the trachea, and thyroid lobes (Figure 5D–G). Palliative chemotherapy with gemcitabine and docetaxel was recommended for the patient.

The progression of the cervical metastases was fulminant, and the patient presented as an emergency with progressive dyspnea and acute respiratory failure due to tracheal compression. Emergency surgical intervention was performed, including en bloc excision of the tumor and right thyroid lobectomy. Histopathological and immunohistochemical examinations confirmed a high-grade spindle cell mesenchymal tumor compatible with a metastatic malignant phyllodes tumor, with heterogeneous CD34 positivity (Figure 3E–G).

The postoperative course was favorable, with no respiratory complications. The patient was discharged in good general condition and continued palliative chemotherapy, with a planned MRI re-evaluation after the fourth cycle.

At 15 months of follow-up, the patient developed progressive left lateral cervical swelling associated with worsening dyspnea. Cervical computed tomography revealed a large, multilobulated proliferative mass located in the anterior and left lateral cervical regions, containing non-critical internal necrotic areas, with significant narrowing and rightward displacement of the trachea. Additionally, a right submandibular lymph node measuring 1.6 cm with a necrotic center was observed. Chest computed tomography revealed a metastatic lesion measuring 3.0 × 2.8 cm in the lower lobe of the right lung (Figure 5H,I).

Emergency tracheostomy was performed due to acute respiratory failure caused by tracheal compression. Given the advanced stage of the disease and the patient’s deteriorating general condition, management was limited to surveillance and supportive care. Despite these measures, the patient’s clinical status continued to decline, ultimately resulting in death.

## 3. Discussion

Malignant phyllodes tumors (MPTs) are rare fibroepithelial neoplasms of the breast, accounting for less than 1% of all breast tumors and approximately 10–20% of all phyllodes tumors [1,2,3,4]. Despite their rarity, MPTs exhibit aggressive biological behavior, with reported local recurrence rates of up to 40% and distant metastases occurring in 9–27% of cases [3,5]. Once metastatic disease develops, the prognosis is poor, with a median overall survival typically ranging between 6 and 24 months [5,14].

The present case is remarkable for its rare constellation of aggressive features, including a giant, long-standing ulcerated malignant phyllodes tumor, followed by cervical lymph node metastasis, direct invasion of the laryngeal and thyroid cartilages, secondary thyroid gland involvement, and severe tracheal compression leading to acute respiratory failure. Such evolution is exceedingly uncommon and highlights the unpredictable dissemination patterns and life-threatening potential of malignant phyllodes tumors.

### 3.1. Tumor Size, Histology, and Aggressive Behavior

Giant phyllodes tumors, generally defined as lesions measuring ≥10 cm or weighing more than 1 kg, are uncommon but are strongly associated with malignancy and poor outcomes [13,15,16]. Several studies have demonstrated a clear correlation between tumor size and its metastatic potential. Reinfuss et al. and Kapiris et al. identified tumor size greater than 10 cm as a significant independent predictor of distant metastasis and reduced survival [16,17,18].

In the present case, the tumor weighed over 5 kg and demonstrated marked stromal overgrowth, a mitotic index exceeding 15 mitoses per 10 high-power fields, and a Ki-67 proliferation index of approximately 40%, all of which are recognized predictors of aggressive clinical behavior and unfavorable prognosis [1,13,19]. Chronic ulceration and delayed presentation may have facilitated early hematogenous dissemination before definitive surgical treatment.

### 3.2. Metastatic Patterns and Lymph Node Involvement

Malignant phyllodes tumors characteristically metastasize via the hematogenous route, reflecting their sarcomatous biology. The lungs represent the most frequent metastatic site (60–80%), followed by bone, liver, and brain [5,9,11]. In contrast, lymph node involvement is distinctly rare, reported in fewer than 1–5% of cases, and is often reactive rather than metastatic [7,12]. Consequently, routine axillary lymph node dissection is not recommended in the absence of clinically suspicious lymphadenopathy [1,20,21,22].

Nevertheless, an increasing number of case reports and small series have documented true lymphatic dissemination in high-grade malignant phyllodes tumors [7,8,10,11,12]. Cervical lymph node metastasis remains exceptional and has only been sporadically reported. Even more unusual is the direct invasion of adjacent cervical structures, such as the thyroid gland and laryngeal cartilages. Thyroid metastasis or invasion from breast phyllodes tumors has been documented only in isolated case reports. Giorgadze et al. reported the first case of a malignant phyllodes tumor metastasizing to a thyroid Hürthle cell adenoma, representing a rare example of tumor-to-tumor metastasis. Histologically, the metastatic lesion showed spindle-cell sarcomatous features identical to those of the primary breast tumor and lacked expression of thyroid-specific markers, confirming its secondary nature [23]. Subsequently, Kho and Abelardo described an isolated thyroid metastasis arising from a benign phyllodes tumor, underscoring the biological behavior of phyllodes tumors and demonstrating that even histologically benign lesions may give rise to distant hematogenous metastases years after the initial treatment [24]. In the reported cases, thyroid metastasis typically presents as a rapidly enlarging thyroid mass, often clinically and radiologically mimicking primary thyroid neoplasms. Thyroid involvement generally reflects aggressive tumor biology or advanced disease and is associated with a poor prognosis, with surgical treatment usually performed for palliative purposes, particularly in the presence of compressive or airway symptoms. The present case expands the known metastatic spectrum of malignant phyllodes tumors by demonstrating aggressive anterior cervical dissemination with secondary thyroid involvement and critical airway compromise, culminating in fatal respiratory failure.

### 3.3. Surgical Management and Adjuvant Therapy

Complete surgical excision with negative margins remains the cornerstone of treatment for malignant phyllodes tumors [1,20,21]. Current guidelines recommend wide local excision with margins of at least 1 cm or mastectomy when adequate margins cannot be achieved. Axillary lymph node dissection is reserved for cases with clinically or radiologically suspicious nodes [1,20].

Adjuvant radiotherapy has been shown to significantly reduce local recurrence rates in high-risk malignant phyllodes tumors, particularly in cases with large tumor size, close or positive margins, or recurrent disease [19,21]. However, no consistent survival benefits have been demonstrated. In the present case, postoperative radiotherapy achieved local control but failed to prevent rapid systemic progression, underscoring the limited impact of locoregional treatments once an aggressive metastatic disease is established.

### 3.4. Systemic Therapy and Molecular Perspectives

Systemic chemotherapy for malignant phyllodes tumors is largely extrapolated from soft-tissue sarcoma protocols and remains predominantly palliative [14]. Anthracycline- and ifosfamide-based regimens may induce partial responses; however, these are typically transient, with limited progression-free survival [14]. In particular, adjuvant chemotherapy following complete surgical resection has not demonstrated a significant benefit, as shown in retrospective analyses, and is therefore not routinely recommended [16]. The short-lived response observed in the present case is consistent with the published data.

Recent advances in molecular profiling have revealed recurrent genetic alterations in malignant phyllodes tumors involving the stromal component, which drive tumor progression and metastatic potential. One of the most frequently reported alterations across all grades of phyllodes tumors is the MED12 exon 2 mutation, which is shared with fibroadenomas and is thought to represent an early tumorigenic event [25]. However, MED12 mutations are less frequent in malignant tumors than in benign lesions, suggesting a reduced role in late-stage progression [26,27].

Malignant transformation is associated with additional genomic instability and oncogenic alterations involving *TP53*, *RB1*, *EGFR*, *PIK3CA*, *NF1* alterations, *TERT* promoter regions, and rare but potentially actionable kinase fusions involving *NTRK*, *FGFR*, and *BRAF* [22,28,29,30,31,32]. *TERT* promoter mutations, in particular, show a strong association with borderline and malignant phyllodes tumors and are considered key events in tumor progression and aggressive behavior of the tumor. The coexistence of *MED12* and *TERT* promoter mutations has been linked to a higher tumor grade and recurrence risk [33]. Copy number alterations are more prevalent in malignant tumors and include gains in chromosomes 1q, 5p, 7, and 8, and losses in 13q and 9p, reflecting increasing chromosomal instability with tumor grade [25,32]. Overexpression or amplification of EGFR has also been reported, supporting its potential therapeutic relevance, although targeted therapies remain investigational. Overall, malignant phyllodes tumors exhibit a sarcoma-like molecular profile, supporting their aggressive clinical behavior and limited response to conventional breast cancer therapy.

These findings support the early use of comprehensive next-generation sequencing, including RNA-based fusion analysis, particularly in advanced or refractory cases, to identify patients who are candidates for targeted therapies [31,32].

Immunotherapy remains investigational and is currently experimental in malignant phyllodes tumors. Most malignant phyllodes tumors exhibit low tumor mutational burden and limited PD-L1 expression; however, isolated reports and broader sarcoma immunotherapy trials, such as SARCO28, suggest that selected patients may benefit from combined immunotherapeutic or antiangiogenic approaches [14,28,34].

### 3.5. Prognostic Implications and Surveillance

Adverse prognostic factors consistently reported in the literature include tumor size greater than 10 cm, stromal overgrowth, high mitotic activity, positive surgical margins, heterologous differentiation, and early recurrence [13,16,18]. Locoregional recurrence has been shown to strongly predict subsequent distant metastasis [13,14,35]. In the present case, rapid dissemination within three months of definitive treatment placed the patient within a very high-risk subgroup characterized by extremely poor survival outcomes.

The unusual cervical and thyroid involvement observed in this case has important implications for postoperative surveillance. In patients with giant tumors or aggressive histological features, systematic evaluation of the cervical and supraclavicular regions should be incorporated into follow-up protocols, even in the absence of local breast recurrence.

## 4. Conclusions

Malignant phyllodes tumors can exhibit exceptionally aggressive biological behavior, characterized by a high potential for atypical metastasis, including cervical lymph node dissemination and invasion of adjacent structures such as the thyroid and trachea. These particularities highlight the importance of a personalized therapeutic approach and constant clinical monitoring of patients. Radical surgical treatment remains the cornerstone in the management of these cases; however, the limited effectiveness of chemotherapy and radiotherapy in metastatic forms underscores the need to identify and develop modern targeted therapies based on the tumor’s molecular profile. Careful multidisciplinary monitoring, complemented by periodic imaging studies of the cervical, thoracic, and abdominal regions, is essential for early detection of recurrence and distant metastases. This contributes to optimizing the prognosis and gaining a better understanding of the biological variability of these rare tumors.

## Data Availability

The original contributions presented in this study are included in this article as Supplementary Material. Further inquiries should be directed to the corresponding author.

## Supplementary Materials

The following supporting information can be downloaded at: https://www.mdpi.com/article/10.3390/diseases14030114/s1, Table S1: Timeline of clinical course and management of the patient with malignant phyllodes tumor.

## Abbreviations

The following abbreviations are used in this manuscript.

PTs	Phyllodes tumors
WHO	World Health Organization
BMI	Body Mass Index
HE	Hematoxylin and eosin
Ki-67	Ki-67 antigen
CD-34	Cluster of Differentiation 34
MRI	Magnetic resonance imaging
PET-CT	Positron Emission Tomography—Computed Tomography
MPTs	Malignant phyllodes tumors
*MED12*	*Mediator Complex Subunit 12*
*TP53*	*Tumor Protein p53*
*RB1*	*Retinoblastoma 1*
*EGFR*	*Epidermal Growth Factor Receptor*
*PIK3CA*	*Phosphatidylinositol-4,5-Bisphosphate 3-Kinase Catalytic Subunit Alpha*
*NF1*	*Neurofibromin 1*
*TERT*	*Telomerase Reverse Transcriptase*
*NTRK*	*Neurotrophic Tyrosine Receptor Kinase*
*FGFR*	*Fibroblast Growth Factor Receptor*
*BRAF*	*B-Rapidly Accelerated Fibrosarcoma*
RNA	Ribonucleic Acid
PD-L1	Programmed Death-Ligand 1
SARCO28	Sarcoma 28-gene panel
