# Supplementary material for: Giant Malignant Phyllodes Tumor with Secondary Thyroid Involvement
Source: Diseases. 2026 Mar 22;14(3):114. doi: 10.3390/diseases14030114 (PMC13024844; doi:10.3390/diseases14030114)
Supplement: Supplementary file 1 [file diseases-14-00114-s001.zip › diseases-4155553-supplementary.pdf]

Table S1. Timeline of clinical course and management of the patient with malignant phyllodes tumor.

Time	Clinical Event / Intervention	Investigations and Findings	Treatment / Management	Outcome / Evolution
Initial presentation	46-year-old female, cachectic with a giant ulcerated left-breast mass (~2 years).	US + core-needle biopsy → Diagnostic: malignant phyllodes tumor (MPT); mild infection; anemia + thrombocytosis.	Improved general condition; infection controlled; transfusion (Hb normalized ≥ 8 g/dL);surgery scheduled.	
Week 2- Surgery	Radical left mastectomy with level I–II axillary dissection.	Gross specimen = ulcerated fibroepithelial tumor 5.2 kg. Histopathology: malignant phyllodes tumor, pT4aN0 (0/6), 15 mitoses/10 HPF, Ki-67 ≈ 40%, CD34 +.	Definitive surgery performed uneventfully.	Post-op recovery favorable; normalization of labs; discharge + oncologic referral.
Weeks 4–10 (post-op)	Post-surgical consolidation phase.	No signs of infection; healed surgical wound.	Adjuvant radiotherapy: 50 Gy in 25 fractions .	Well tolerated; patient clinically stable.
Month 3 after RT	New right-lateral cervical swelling, dysphonia.	Neck US + MRI: bilobed cervical mass (~4 × 4 cm) invading right thyroid cartilage/laryngeal wall;<br>PET-CT → FDG-avid cervical nodes + pulmonary foci.	Core-needle biopsy → sarcomatoid metastatic lymph node compatible with MPT recurrence. Port-a-Cath inserted.	Diagnosis: metastatic malignant phyllodes tumor.
Months 4–8 after surgery	Systemic palliative therapy initiated.	Mild anemia and elevated liver enzymes.	Chemotherapy: LT-AIM (adriamycin + ifosfamide + mesna) × 6 cycles<br>Dose adjustments for thrombocytosis, vit B12/folate deficiency.	Partial response: major shrinkage of cervical and pulmonary lesions.
Month 9	Re-staging imaging.	MRI/PET-CT: residual right cervical nodes 1.8 - 2.1 cm; no new lesions.	Surveillance; supportive therapy.	Temporary disease control.
Month 10–11	Recurrence/ progression	MRI: growth of cervical mass (4.6 × 4.2 × 5.0 cm) with thyroid cartilage and tracheal invasion.	Second-line palliative chemotherapy : Gemcitabine (days 1, 8) + Docetaxel (day 8).	Rapid progression with airway compression.
Month 11 (Emergency)	Acute dyspnea – respiratory distress from tracheal compression.	CT neck: giant anterior cervical tumor (7.9 × 8.3 × 8.9 cm), trans-spatial invasion, thyroid lysis, vascularized mass.	Emergency surgery: en bloc excision + right lobectomy thyroid.<br>Airway decompression achieved.	Patient recovered post-op.
Post-emergency surgery	Pathological confirmation of recurrence.	Mesenchymal spindle-cell tumor identical to stromal component of primary MPT;<br>CD34 +, SMA –, CKHMW - .	Post-operative antibiotics, analgesics, anticoagulants, nutritional support.	Good immediate recovery; patient discharged.
Months 12–14	Oncologic follow-up.		Palliative chemotherapy maintained: Gemcitabine 100 mg/m² (days 1, 8) + Docetaxel 75 mg/m² (day 8)	First month tolerating therapy well.
Month 15	Left lateral cervical swelling and dyspneea	CT neck scan : Large, multilobular proliferative formation with non-critical internal areas, anterior and left lateral cervical, with narrowing and displacement of the trachea to the right, right submandibular	Tracheostoma for respiratory faillure<br>Surveillance; supportive therapy.	Progressive clinical deterioration culminating in death.

		adenopathy measuring 1.6 cm with necrotic center.<br>Chest CT scan - 3/2.8 cm metastasis in the lower right lobe of the lung.		

Legend / Abbreviations. HPF – High-Power Field; Hb – hemoglobin; RT – radiotherapy; LT-AIM – long-term adriamycin + ifosfamide + mesna protocol; US – ultrasound; PET-CT – positron-emission tomography–computed tomography.

.
